# Pemetrexed for advanced non-small cell lung cancer patients with interstitial lung disease

**DOI:** 10.1186/1471-2407-14-508

**Published:** 2014-07-10

**Authors:** Motoyasu Kato, Takehito Shukuya, Fumiyuki Takahashi, Keita Mori, Kentaro Suina, Tetsuhiko Asao, Ryota Kanemaru, Yuichiro Honma, Keiko Muraki, Koji Sugano, Rina Shibayama, Ryo Koyama, Naoko Shimada, Kazuhisa Takahashi

**Affiliations:** 1 Department of Respiratory Medicine, Juntendo University Graduate School of Medicine, 2-1-1, Hongo, 113-8421 Bunkyo-ku, Tokyo, Japan; 2 Clinical Trial Coordination Office, Shizuoka Cancer Center, 1007 Shimonagakubo, Nagaizumi-cho, 411-8777 Suntou-gun, Shizuoka, Japan

**Keywords:** Non-small cell lung cancer, Pemetrexed, Interstitial pneumonitis, Idiopathic pulmonary fibrosis, Acute lung injury, Acute exacerbation

## Abstract

**Background:**

Non-small cell lung cancer (NSCLC) patients with interstitial lung disease (ILD) need to be approached carefully given the high incidence of pulmonary toxicity. Pemetrexed (PEM) is the key drug for the treatment of NSCLC. However, its safety, especially with respect to the exacerbation of ILD, and efficacy in NSCLC patients with ILD have yet to be established.

**Method:**

We investigated the safety and efficacy of PEM monotherapy in NSCLC patients with or without idiopathic interstitial pneumonia (IIPs). The medical charts of these patients were retrospectively reviewed.

**Results:**

Twenty-five patients diagnosed as having IIPs (IIPs group) and 88 patients without ILD (non-ILD group) were treated with PEM monotherapy at Juntendo University Hospital between 2009 and 2013. In the IIPs group, 12 patients were found to have usual interstitial pneumonitis (UIP) on chest computed tomography (CT) (UIP group) and the other 13 patients showed a non-UIP pattern on chest CT (non-UIP IIPs group). Three patients in the IIPs group (2 in the UIP group and 1 in the non-UIP IIPs group) and 1 in the non-ILD group developed pulmonary toxicity during treatment (3.5% overall, 12.0% in the IIPs group versus 1.1% in the non-ILD group). Moreover, all 3 patients in the IIPs group died of pulmonary toxicity. Overall survival tended to be longer in the non-ILD group than in the IIPs group *(p = 0.08*). Multivariate analyses demonstrated that IIPs was the only significant independent risk factor for PEM-related pulmonary toxicity.

**Conclusion:**

We found that the incidence of PEM-related pulmonary toxicity was significantly higher amongst NSCLC patients with IIPs than among those without IIPs. Particular care must be taken when administering PEM to treat NSCLC patients with IIPs.

## Background

Lung cancer is a pulmonary disease with a poor prognosis, and is frequently associated with interstitial lung disease (ILD), especially idiopathic interstitial pneumonitis (IIPs). ILD consists of disorders of known causes as well as disorders of unknown cause. IIPs are most frequent disease in ILD. Then, ILDs except for IIPs contains many heterogeneous diseases like collagen vascular disease related interstitial pneumonia, sarcoidosis, pneumoconiosis, radiation pneumonitis and drug related lung injury.

The incidence of lung cancer in patients with ILD has been reported to be approximately 15–30%, [1] and its incidence at autopsy in Japanese patients with usual interstitial pneumonitis (UIP) has been reported to be 48.2% (40 cases in total autopsies) [2]. On the other hand, it has been reported that the prevalence of idiopathic pulmonary fibrosis (IPF) in the United States and Japan were estimated to be 14.0 to 42.7 and 3.44 per 100,000 people, respectively [3,4]. It is unknown whether the prevalence of IPF and prevalence of lung cancer in IPF patients are influenced by ethnic, geographic or cultural factors or not, because there are no reports on direct comparison of incidence and prevalence rate between Caucasian and Japanese [5].

Non-small cell lung cancer (NSCLC) is a progressive disease, and hence, NSCLC patients are usually diagnosed with advanced stage cancer and usually receive chemotherapy. Acute lung injury (ALI) and exacerbation of ILD are known common side effects of chemotherapy, for which pre-existing pulmonary fibrosis is reported to be the most significant risk factor. Furthermore, NSCLC patients with ILD were found to have a greater risk of developing pulmonary toxicity (ALI/exacerbation of ILD) as a result of chemotherapy than patients without ILD, in a prospective study conducted in Japan [6]. Further, the incidence of exacerbation of ILD due to chemotherapy is significantly higher among lung cancer patients with a UIP pattern on CT findings than among those with a non-UIP pattern [7]. However, it is unclear which regimen and anticancer agent presents the lower or higher risk of pulmonary toxicity for NSCLC patients with ILD.

It has been reported that the incidence of pulmonary toxicity in Japanese patients (2%) is higher than in USA patients (0.3%) in treatment of gefitinib for NSCLC patients by FDA [8]. Although there are no reports on direct comparison of pulmonary toxicity induced by cytotoxic agents among Japanese, non-Asian and Caucasian, the incidence of docetaxel (DTX)-induced pulmonary toxicity is about 2.1% (6 cases in 276 total patients) in Caucasian [9] and 4.6% (18 cases in 392 total patients) [10] in Japanese. And it has been reported that the incidence of bleomycin-induced lung injury was 0.66% in Japan and 0.01% in global cases [11]. Based on these reports, chemo-associated pulmonary toxicity seemed to be more frequent in Japanese patients than Caucasian patients, and this ethnic difference may be explained by genetically. However, so far, there is no clear scientific evidence which reveal this ethnic difference.

Pemetrexed (PEM) is an established multi-targeted anti-folate drug and one of the important anticancer agents for advanced non-squamous NSCLC (NSqNSCLC) and malignant pleural mesothelioma (MPM). PEM combined with platinum agents is often used as a first-line chemotherapeutic agent to treat patients with advanced NSqNSCLC and MPM [12]. PEM monotherapy is also an effective second-line treatment for patients with advanced NSqNSCLC. Hanna et al. studied the efficacy and safety of PEM monotherapy in American NSqNSCLC patients and found that the incidence of PEM-induced pulmonary toxicity was approximately 0.8% (2 cases in total 265 patients), [9] although this was found to be slightly higher (3.5%, 4 cases in total 114 cases) among Japanese NSCLC patients [13]. Amongst MPM patients, Kuribayashi et al. found that the incidence of PEM and cisplatin (CDDP)-related pulmonary toxicity was 0.9% (8 cases in total 903 patients) [14]. However, the incidence of PEM-related pulmonary toxicity in NSCLC patients with IIPs or IPF is yet to be established. In the study reported here, we compared the efficacy and safety (with special regard to pulmonary toxicity) of PEM treatment in advanced NSCLC patients with IIPs to that in patients without ILD in Japan.

## Methods

### Patient selection

**Figure 1:**
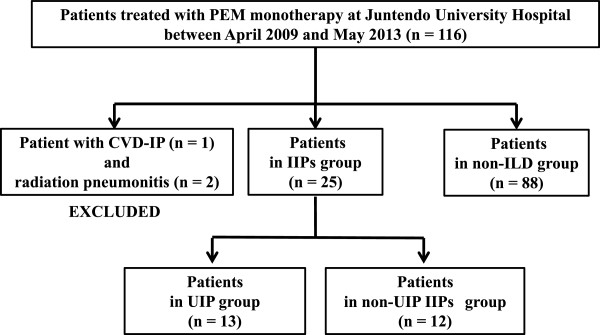
Study patients.

Between April 2009 and May 2013, 116 NSCLC patients were administered PEM monotherapy at Juntendo University Hospital. Two patients with radiation pneumonitis and 1 patient with collagen vascular disease associated with interstitial pneumonitis were excluded from this study. There were no patients with other known causes of ILD (e.g., sarcoidosis, pneumoconiosis, and chronic hypersensitivity pneumonitis). Twenty-five NSCLC patients diagnosed as having IIPs (IIPs group) and 88 patients without ILD, including IIPs (non-ILD group) were enrolled in this retrospective cohort study (Figure 1).

Patients found to have an interstitial shadow on a chest CT scan were enrolled into the IIPs group, and those without an interstitial shadow were entered into the non-ILD group. Interstitial shadows were defined as reticular shadow, ground glass opacity, honeycombing, and traction bronchiectasis. The IIPs group was further divided into patients with honeycombing with or without traction bronchiectasis; subpleural, basal predominance; or reticular abnormality on chest CT (UIP group) and those with an interstitial shadow without honeycombing on chest CT (non-UIP IIPs group). The UIP pattern was diagnosed based on chest CT features as defined by “An Official ATS/ERS/JRS/ALAT Statement: Idiopathic Pulmonary Fibrosis: Evidence-based Guidelines for Diagnosis and Management” [5]. Three pulmonologists (MK, TS and RK) reviewed pretreatment CT and plain X-ray films of the chest. All patients involved in this trial provided informed consent for use of medical data. This study protocol was approved by Juntendo University Ethical Committee and registered under number is 25–408.

### Treatment method

Patients were administered 500 mg/m^2^ PEM as a 10-minute intravenous infusion every 3 weeks. Before the treatment cycle could be started, patients needed to have an absolute neutrocyte count (ANC) of at least 1,500/mm^3^, a platelet count of at least 100,000/mm^3^, transaminase values less than 2.5 times the upper limit of the normal range, and total serum bilirubin and creatinine levels less than 1.5 times the upper limit of the normal range. Patients in the PEM arm were asked to take a daily oral folic acid dose of 500 μg beginning approximately one week before the first dose of PEM and continuing until 3 weeks after the last dose of PEM. Vitamin B12 (1 g) was administered by intramuscular injection approximately 1 week before the first dose of PEM and was repeated approximately every 9 weeks until discontinuation of PEM [9,13].

### Evaluation of response and toxicity

Response Evaluation Criteria in Solid Tumor (RECIST) version 1.1 was used to evaluate the response to treatment. Chest CT was performed after every 2 cycles of PEM in order to evaluate the change in tumor size. Adverse events were evaluated until 4 weeks after the completion of chemotherapy according to Common Terminology Criteria for Adverse Event (CTCAE) version 4.0. Pneumonitis, pulmonary fibrosis, and adult respiratory distress syndrome in CTCAE term were defined as pulmonary toxicity. We evaluated and compared response, survival, and toxicities between the IIPs and non-ILD groups, and in order to evaluate the response and pulmonary toxicity, we also analyzed patients in both the UIP and non-UIP IIPs groups.

### Statistical method

We used the Chi square test, Fisher’s exact test, or Wilcoxon two-sample test to compare patient characteristics, response to PEM, and the frequency of toxicities, as appropriate. Logistic regression analysis was used to estimate the risk of pulmonary toxicity. Progression-free survival (PFS) and overall survival (OS) curves were plotted using the Kaplan Meier method and the differences in PFS and OS between IIPs group and non-ILD group were analyzed using the log-rank test. Univariate and multivariate analyses were performed in order to identify risk factors for PEM-related pulmonary toxicity. Multivariate analyses were performed using logistic regression to assess the relationship between various factors and pulmonary toxicity.

All *p-*values less than 0.05 were considered statistically significant. All statistical analyses were performed using JMP ver. 8.0 for Windows (SAS Institute Inc., Cary, NC, USA).

## Results

### Patient characteristics

Between April 2009 and May 2013, 25 NSCLC patients in the IIPs group and 88 patients in the non-ILD group were administered PEM monotherapy at Juntendo University Hospital and enrolled in this retrospective cohort study. Of the patients in the IIPs group, 12 with an interstitial shadow and honeycombing on a chest CT scan were entered into the UIP group and 13 patients with an interstitial shadow but without honeycombing were entered into the non-UIP IIPs group. The baseline characteristics of all patients and their diagnoses are listed in Table 1. There were no significant differences in age, performance status (PS), disease stage, or tumor histology. There was, however, a significant difference in the gender distribution between the IIPs and non-ILD groups (23 [92%] versus 41 [46.6%] male patients, *p = 0.0001*). Twenty-four (96%) and 49 patients (55.7%) had a smoking history in the IIPs and non-ILD group, respectively (*p = 0.001*). Three patients in the IIPs group (12.0%) had the sensitive epithelial growth factor receptor (EGFR) mutation (1 in the UIP group [8.3%] and 2 in non-UIP IIPs group [15.3%]) compared to 28 in the non-ILD group (31.8%; *p = 0.088*). A higher proportion of patients in the non-ILD group than in the IIPs group carried the sensitive EGFR mutation, although this difference was not statistically significant.

**Table 1:** Patient characteristics

		**IIPs**			**Non-ILD**
		**UIP + non- UIP IIPs**	**UIP**	**Non-UIP IIPs**	
**Number of patients**		**25**	**12**	**13**	**88**
**Age (year)**	Median (range)	69 (58–81)	71 (58–80)	68 (60–81)	70 (35–92)
**Gender**	Male (%)	23 (92.0)	12 (100)	11 (84.6)	41 (46.6)
**Smoking History**	Yes (%)	24 (96.0)	12 (100)	12 (92.3)	49 (55.7)
**Performance status**	0-1 (%)	21 (84.0)	10 (83.3)	11 6)	80 (92.0)
**Histology**	Adenocarcinoma (%)	22 (88.0)	10 (83.3)	12 (92.3)	83 (94.3)
**Stage**	IIIB + IV (%)	19 (76.0)	10 (83.3)	9 (69.2)	73 (82.9)
**Line**	1/2/3	5/10/10	2/4/6	3/6/4	13/44/31
**EGFR mutation**	Sensitive (%)	3 (12.0)	1 (8.3)	2 (15.3)	28 (31.8)

### Efficacy and survival

**Figure 2:**
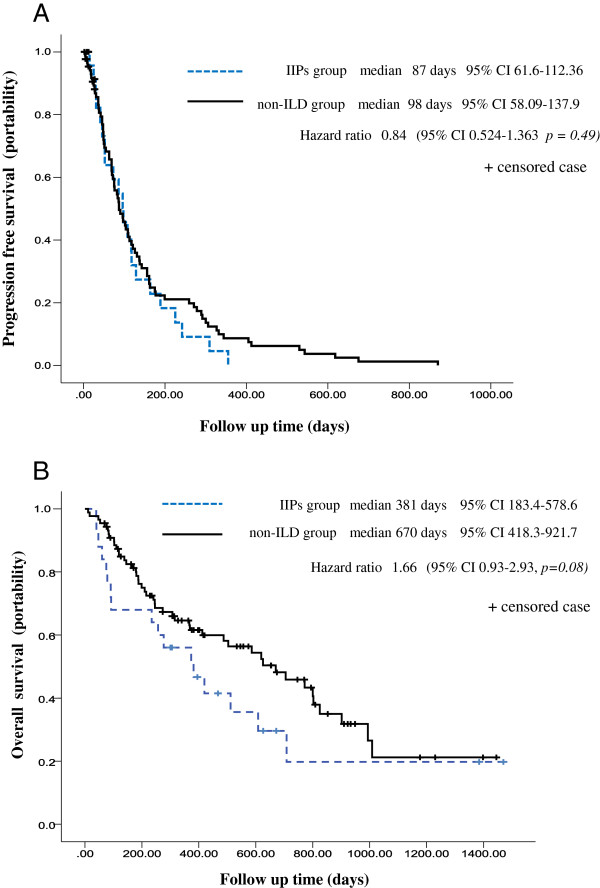
**Kaplan–Meier curves of progression-free survival and overall survival.  (A)** Kaplan–Meier plot of progression-free survival for patients with IIPs and non-ILD. **(B)** Kaplan–Meier plot of overall survival for patients with IIPs and non-ILD.

Treatment response and outcome was not relatively different between two groups. The response rates did not differ significantly (12.0% in the IIPs group versus 18.1% in the non-ILD group and 8.3% in the UIP group versus 7.7% in the non-UIP IIPs group). Responses of 2 patients in the IIPs group and 1 patient in the non-ILD group were not evaluable. PFS also did not differ significantly between the IIPs groups and the non-ILD group (median, 87 days in the IIPs groups versus 98 days in the non-ILD groups; hazard ratio, 0.84; 95% confidence interval [CI], 0.52–1.36; *p = 0.49*; Figure 2A). Further, although there was no significant difference in OS between the 2 groups (median, 381 days in the IIPs group versus 670 days in the non-ILD group; hazard ratio, 1.66; 95% CI, 0.93–1.93; *p = 0.08*), OS tended to be longer in the non-ILD group (Figure 2B). The disease control rates differed significantly between the groups (48.0% in the non-ILD group versus 72.7% in the IIPs group, *p = 0.03* and 25% in the UIP group versus 61.5% in the non-UIP IIPs group, *p = 0.03*).

### Toxicity

All patients were assessable for toxicities. The principal grade 3 or 4 toxicities, with the exception of pulmonary toxicity, are summarized in Table 2. There were no significant differences in grade 3 or 4 toxicities between the 2 groups.

**Table 2:** Toxicities (excluded for pulmonary toxicities)

	**IIPs**		**non-ILD**		
**Number of patients**	**25**		**88**		*P*
	**n**	**%**	**n**	**%**	
**AST, ALT**	1	4	2	2.2	*0.45*
**Eruption**	4	16	4	4.4	*0.13*
**Nausea**	1	4	3	3.4	*0.63*
**Allergic fever**	0	0	4	4.4	*0.63*
**Neutropenia**	3	12	14	15.9	*0.85*
**Leukocytopenia**	2	8	7	8	*0.68*
**Anemia**	0	0	2	2.2	*0.92*
**Thorombocytopenia**	0	0	1	1.1	*0.50*
**Febrile neutrocytopenia**	0	0	1	1.1	*0.50*

### Pulmonary toxicity

Pulmonary toxicity was experienced by 2 patients in the UIP group, and a single patient each in the non-UIP IIPs and non-ILD groups, making the overall incidence of pulmonary toxicity associated with PEM 3.5% (Table 3). The incidence of PEM-related pulmonary toxicity was significantly higher in the IIPs group than in the non-ILD group (12.0% versus 1.1%; odds ratio [OR], 11.8; 95% CI, 1.17–119; *p = 0.03*), and the incidence of pulmonary toxicity tended to be higher in the UIP group than in the non-UIP IIPs group (16.7% versus 7.7%; OR, 7.25; 95% CI 0.42–123.69; *p = 0.59*). The number of cycles between the first PEM treatment and the occurrence of pulmonary toxicity was 3 in 1 case, 2 in 2 cases, and 1 in 1 case. Chest CT findings at the onset of toxicity in the 2 cases of PEM-related pulmonary toxicity in the IIPs group (a single patient each in the UIP and non-UIP IIPs groups) showed a diffuse alveolar damage (DAD) pattern. The DAD pattern consists of new diffuse and bilateral ground glass opacity (GGO) together with a reticular shadow in the non-segmental predominance of lung opacity with new traction bronchiectasis [15,16]. However, the chest CT findings at the onset of pulmonary toxicity in 2 other patients (a single patient each in the UIP and in the non-ILD groups) revealed a hypersensitivity pneumonitis (HP) pattern (only new GGO) [15,16]. All 3 patients in the IIPs group and no patients in non-ILD group died of pulmonary toxicity. Three patients in the IIPs group received steroid pulse therapy after the diagnosis of pulmonary toxicity. A single patient with DAD discovered on chest CT in the non-UIP IIPs group died of respiratory failure 2 weeks after the initiation of steroid pulse therapy, and another patient with HP discovered on chest CT in the UIP group was administered 500 mg/day methylprednisolone for 3 days with a gradually reduced dose of oral prednisolone after steroid pulse therapy. However, in this latter case, ILD exacerbation recurred when oral prednisolone was administered at the dose of 20 mg, and chest CT revealed a DAD pattern. Although the patient was administered 1 g of methylprednisolone, he died 5 days after the initiation of the second steroid pulse therapy. A further patient in the UIP group recovered from exacerbation of ILD after steroid pulse therapy but died of pneumonitis and respiratory failure 3 months after the onset of this toxicity. Conversely, for a patient in the non-ILD group, cessation of PEM therapy alone resulted in an improvement in symptoms and image findings after 1 week. In all cases, we excluded bacterial pneumonia, pulmonary embolism, and heart failure by physical examination, laboratory and culture findings, or echocardiography. The results of univariate and multivariate analyses of risk factors for pulmonary toxicity associated with PEM therapy are shown in Tables 4 and 5 IIPs was significantly associated with pulmonary toxicity (OR, 11.8; 95% CI, 1.17–119.6; *p = 0.03*). Multivariate analyses were performed using six variables (age, gender, smoking history, performance status, number of treatment lines, and the presence of IIPs) and revealed that only IIPs (OR, 34.37; 95% CI, 1.64–45566.21; *p = 0.019*) was a significant independent risk factor.

**Table 3:** Pulmonary toxicity

			**IIPs**				**Non-IIPs**		**Total**	
	**UIP + non- UIP IIPs**		**UIP**		**Non-UIP IIPs**					
	**n**	**%**	**n**	**%**	**n**	**%**	**n**	**%**	**N**	**%**
**Number of Patients**	25		12		13		88		113	
**Pulmonary toxicity**	3	12	2	16.7	1	7.7	1	1.1	4	3.5
**Grade 5 ILD**	3		2		1		0		3	

**Table 4:** Univariate analysis of risk factors associated with PEM-related pulmonary toxicity

		**Number of patients**				
	**Overall**	**Pulmonary toxicity**	**non-Pulmonary toxicity**	**Odds ratio**	**95% CI**	***P***
**Number of patients**	113	4	109			
**Age (year)**				0.36	0.03-3.62	*0.62*
**<71**	60	3	57			
**≧71**	53	1	52			
**Gender**				2.36	0.23-23.41	*0.63*
**Female**	49	1	48			
**Male**	64	3	61			
**Smoking history**				1.80	0.18-17.9	*1.00*
**No**	42	1	41			
**Yes**	71	3	68			
**Performance status**				0.34	0.28-31.0	*0.37*
**0-1**	101	3	98			
**2-3**	11	1	10			
**Number of line**				0.57	0.05-5.71	*1.00*
**1-2**	72	3	69			
**3-**	41	1	40			
**IIPs**				11.8	1.17-119.6	0.03*
**No**	88	1	87			
**Yes**	25	3	22			

*Statistically significant by Fisher's exact test.

**Table 5:** Multivariate Analysis of Risk Factors Associated with PEM-induced ILD

	**Odds ratio**	**95% CI**	***P***
**Variable**			
**Age (<71 vs ≧71)**	0.20	0.003-2.52	*0.23*
**Gender (Female vs Male)**	1.32	0.03-53.99	*0.86*
**Smoking history (no vs yes)**	4.68	0.09-443.12	*0.44*
**Performance status (0–1 vs 2–3)**	0.17	0.005-5.38	*0.28*
**Number of line (1–2 vs 3-)**	0.23	0.009-2.83	*0.27*
**IIPs (no vs yes)**	34.37	1.64-4566.21	0.019*

*Statistically significant by Logistic regression analysis.

## Discussion

To our knowledge, this is the first study to evaluate and compare the safety and efficacy of PEM monotherapy between NSCLC patients with IIPs and without ILD. Previous reports of PEM-induced pulmonary toxicity are summarized in Table 6[17-22]. There was only a single case of PEM monotherapy-induced pulmonary toxicity. As a result, the risk factors for PEM monotherapy-induced pulmonary toxicity were unclear, especially with regard to the presence of ILD. Our findings indicate that ILD is a risk factor for PEM monotherapy-induced pulmonary toxicity. We suggest therefore that the presence of ILD should be addressed before treating NSCLC patients with PEM monotherapy, as this is frequently found to be coincident with this malignancy. Of the 8 patients in previous studies, treatment with oral steroids improved the symptoms and image findings in 2 cases. Although other patients received intravenous steroid pulse therapy, 4 of them died due to a worsening of respiratory failure. Amongst the cases we report here, 3 patients in the IIPs group were administered steroid pulse therapy, but 2 of them died of respiratory failure and the other recovered from exacerbation of ILD, but died when it subsequently recurred. Only 1 patient, in the non-ILD group, recovered as a result of drug withdrawal alone. It seems therefore that the prognosis after pulmonary toxicity is worse for patients with pre-existing ILD before chemotherapy.

**Table 6:** Previous reports of PEM-induced ILD and our cases

	**A/G**	**Before PEM CT findings**	**Histology**	**Post RTx**	**Pre Chemotherapy**	**Combination therapy**	**No. of cycle**	**Treatment**	**Outcome**	**Reference**
1	65/M	UIP	MPM	No	None	CBDCA	1	Steroid Pulse	Dead	[17] S Sakamoto et al
2	66/F	None	NSCLC (Ad)	No	CDDP + VNR	CDDP	4	Steroid (oral)	Recover	[19] HO Kim. et al
3	71/M	None	MPM	No	None	CDDP	1	Steroid Pulse	Dead	[18] K Nagata. et al
4	77/M	None	MPM	No	None	CBDCA	1	Steroid Pulse	Dead	[18] K Nagata. et al
5	72/F	None	NSCLC (Ad)	No	CBDCA + PEM	CBDCA	2	Steroid (oral)	Recover	[20] B Dhakal. et al
6	64/M	RTx-P	NSCLC (Ad)	Yes	CBDCA + VP-16	None	2	Steroid Pulse	Dead	[21] A Hochstrasser. et al
7	51/M	RTx-P	NSCLC (Ad)	Yes	CDDP + DTX	None	2	Steroid Pulse	Recover	[21] A Hochstrasser. et al. 2012
8	69/M	None	NSCLC (Ad)	No	CBDCA + GEM	None	1	Steroid Pulse	Recover	[22] KH Kim et al
1	64/M	UIP	NSCLC (Ad)	No	CBDCA + PTX + BEV	None	2	Steroid Pulse	Dead	Our case
2	71/M	UIP	NSCLC (Ad)	No	None	None	1	Steroid Pulse	Recover	Our case
3	68/M	GGO	NSCLC (Ad)	No	CBDCA + PTX	None	2	Steroid Pulse	Dead	Our case
4	67/F	None	NSCLC (Ad)	No	CBDCA + PTX, DTX, Gefitinib,	None	3	Drug withdrawn	Recover	Our case

PEM: Pemetrexed, A/G: Age/Gender, UIP: Usual Interstitial Pneumonitis, GGO: Groud Grass Opacity, NSCLC: Non Small Cell Lung Cancer, Ad: Adenocarcinoma, PS: performance Status, RTx: Radiation Therapy, RTX-p: radiation pneumonitis, VNR: Vinorelbine, CBDCA: Calboplatin, PTX: Paclitaxel, BEV: Bevacizmab,VP-16: Etoposide, DTX: Docetaxel, GEM: Gemcitabine, UK: Unknown, MPM: Malignancy Pleural Mesotelioma.

Our results indicate that the overall incidence of PEM-related pulmonary toxicity is 3.5%, which is very similar to the incidence given in previous Japanese reports. [13] However, the incidence of pulmonary toxicity amongst patients with IIPs was 12.0%, and it is particularly noteworthy that the incidence of pulmonary toxicity in patients with a UIP pattern on chest CT was 16.7%. Consistent with these findings, it has been reported that the incidence of the exacerbation of ILD due to chemotherapy was significantly higher amongst lung cancer patients with a UIP pattern on CT than among those with a non-UIP pattern [7].

We evaluated adverse events until 4 weeks after the completion of chemotherapy. Pulmonary toxicity did not occur during 4 weeks after pemetrexed treatment with EGFR-TKI. Pulmonary toxicity also did not occurred during 2 weeks after cessation of pre-treatment EGFR-TKI. Therefore, we suppose PEM-induced pulmonary toxicity was not affected by EGFR-TKI treatment in our research.

As is the case for PEM, DTX monotherapy is frequently used as a second-line treatment for NSCLC. Tamiya et al. reported that the incidence of pulmonary toxicity after DTX therapy was 4.6% (18 cases in 392 total patients), and a pre-existing interstitial change on chest CT was associated with a higher incidence of pulmonary toxicity (25.9%, 17 cases in 68 patients with NSCLC and interstitial shadow on chest CT) [10]. When taken together, these findings indicate that PEM may be suitable as a standard second-line treatment option for NSqNSCLC patients with IIPs. However, it is unclear which regimen and anticancer agent presents the lowest risk of pulmonary toxicity for NSCLC patients with ILD, because most research is retrospective and includes only a relatively small number of patients. A prospective, larger study is warranted to determine the appropriate regimen for advanced NSCLC patients with ILD.

We found no significant difference in PFS with respect to interstitial changes on chest CT, although OS in the non-ILD group tended to be longer than in the IIPs group. One possible explanation for the latter is that more patients had a sensitive EGFR mutation in the non-ILDs group than in the IIPs groups.

This analysis has several limitations. First, the diagnosis of preexisting ILD and exacerbation of ILD was based on chest CT and laboratory findings, and not on histological findings. Second, this study was retrospective, patient characteristics were heterogeneous making it difficult to interpret differences in PFS and OS. However, the onset of pulmonary toxicity is easy to detect, and its frequency and severity might not therefore differ significantly from those found upon prospective evaluation. Third, although the retrospective analysis of pemetrexed-associated pulmonary toxicity is one of the reasonable tools to assess this issue, the small number of patients suffering from ILD also does not allow to make definite conclusions about clinical endpoints such as PFS and OS.

## Conclusion

This is the first study to evaluate and compare the safety and efficacy of PEM monotherapy between NSCLC patients with IIPs and without ILD. Our findings suggest that the risk of pulmonary toxicity is higher for patients with IIPs than those without ILD, and that the risk of pulmonary toxicity might be higher for NSCLC patients with UIP pattern than non-UIP pattern on pretreatment chest CT. We suggest therefore that particular care should be taken when administering PEM to NSCLC patients with IIPs, especially those with a UIP pattern, although the risk of pulmonary toxicity is not significantly higher than when administering other chemotherapeutic agents, for examples DTX.

## Competing interests

The authors have no conflict of interest to declare.

## Authors’ contributions

MK and TS conceived and designed the experiments. TS, FT, RK and KT performed the experiments. MK, TS and KM analyzed the data. MK, TS and RK had reviewed pretreatment CT. KS, TA, RK, YH, KM, KS, RS, RK, and NS contributed for acquisition of clinical data and specimens. TS, FT and TK participated in management and statistical analysis of data. MK wrote the manuscript. TS involved in revising the manuscript. KT provided final approval of the version to be published. All authors read and approved the final manuscript.

## Pre-publication history

The pre-publication history for this paper can be accessed here:

http://www.biomedcentral.com/1471-2407/14/508/prepub
